# Feasibility and safety of low-flow extracorporeal CO_2_ removal managed with a renal replacement platform to enhance lung-protective ventilation of patients with mild-to-moderate ARDS

**DOI:** 10.1186/s13054-018-2038-5

**Published:** 2018-05-10

**Authors:** Matthieu Schmidt, Samir Jaber, Elie Zogheib, Thomas Godet, Gilles Capellier, Alain Combes

**Affiliations:** 1 Sorbonne Université, INSERM, UMRS_1166-iCAN, Institute of Cardiometabolism and Nutrition, Pitié–Salpêtrière Hospital, F-75013 Paris, France; 2 Service de Médecine Intensive et Réanimation, Assistance Publique–Hôpitaux de Paris, Pitié–Salpêtrière Hospital, 47, boulevard de l’Hôpital, F-75013 Paris, France; 3 Département d’Anesthésie et Réanimation B, CHU de Montpellier, Hôpital Saint-Eloi, INSERM Unité 1046, Université Montpellier 1, Montpellier, France; 4 Anesthesiology and Critical Care Medicine Department, Amiens University Hospital, INSERM U-1088, Université de Picardie Jules-Verne, 80054 Amiens Cedex, France; 5 Département de Médecine Périopératoire (MPO), Centre Hospitalier Universitaire (CHU) Clermont-Ferrand, Clermont-Ferrand, France; 6 GReD, UMR/CNRS6293, Université Clermont-Auvergne, INSERM U1103, F-63003 Clermont-Ferrand, France; 7 Medical Intensive Care Unit, Besançon University Hospital, Besançon, France; 8 Research Unit EA 3920 and SFR FED 4234, University of Franche Comté, Besançon, France

**Keywords:** Extracorporeal carbon-dioxide removal, Acute respiratory distress syndrome, Protective ventilation

## Abstract

**Background:**

Extracorporeal carbon-dioxide removal (ECCO_2_R) might allow ultraprotective mechanical ventilation with lower tidal volume (VT) (< 6 ml/kg predicted body weight), plateau pressure (P_plat_) (< 30 cmH_2_O), and driving pressure to limit ventilator-induced lung injury. This study was undertaken to assess the feasibility and safety of ECCO_2_R managed with a renal replacement therapy (RRT) platform to enable very low tidal volume ventilation of patients with mild-to-moderate acute respiratory distress syndrome (ARDS).

**Methods:**

Twenty patients with mild (*n* = 8) or moderate (*n* = 12) ARDS were included. VT was gradually lowered from 6 to 5, 4.5, and 4 ml/kg, and PEEP adjusted to reach 23 ≤ P_plat_ ≤ 25 cmH_2_O. Standalone ECCO_2_R (no hemofilter associated with the RRT platform) was initiated when arterial PaCO_2_ increased by > 20% from its initial value. Ventilation parameters (VT, respiratory rate, PEEP), respiratory system compliance, P_plat_ and driving pressure, arterial blood gases, and ECCO_2_R-system operational characteristics were collected during at least 24 h of very low tidal volume ventilation. Complications, day-28 mortality, need for adjuvant therapies, and data on weaning off ECCO_2_R and mechanical ventilation were also recorded.

**Results:**

While VT was reduced from 6 to 4 ml/kg and P_plat_ kept < 25 cmH_2_O, PEEP was significantly increased from 13.4 ± 3.6 cmH_2_O at baseline to 15.0 ± 3.4 cmH_2_O, and the driving pressure was significantly reduced from 13.0 ± 4.8 to 7.9 ± 3.2 cmH_2_O (both *p* < 0.05). The PaO_2_/FiO_2_ ratio and respiratory-system compliance were not modified after VT reduction. Mild respiratory acidosis occurred, with mean PaCO_2_ increasing from 43 ± 8 to 53 ± 9 mmHg and mean pH decreasing from 7.39 ± 0.1 to 7.32 ± 0.10 from baseline to 4 ml/kg VT, while the respiratory rate was not altered. Mean extracorporeal blood flow, sweep-gas flow, and CO_2_ removal were 421 ± 40 ml/min, 10 ± 0.3 L/min, and 51 ± 26 ml/min, respectively. Mean treatment duration was 31 ± 22 h. Day-28 mortality was 15%.

**Conclusions:**

A low-flow ECCO_2_R device managed with an RRT platform easily and safely enabled very low tidal volume ventilation with moderate increase in PaCO_2_ in patients with mild-to-moderate ARDS.

**Trial registration:**

ClinicalTrials.gov, NCT02606240. Registered on 17 November 2015.

## Background

Over the past few decades, highly significant progress has been made in understanding the pathophysiology of the acute respiratory distress syndrome (ARDS). Recognition of ventilation-induced lung injuries (VILIs) has led to radical modifications of the ventilatory management of these patients [1, 2]. The landmark trial by the ARDSNet group demonstrated that ventilating ARDS patients with a low tidal volume (VT) of 6 ml/kg (vs 12 ml/kg) significantly decreased mortality [3]. However, recent results showed that lung hyperinflation still occurs in approximately 30% of ARDS patients, despite ventilation with the ARDSNet strategy [4]. That analysis suggested a beneficial effect of VT reduction, even for patients already with plateau pressure (P_plat_) < 30 cmH_2_O [5]. Decreasing VT and P_plat_ will also lower the driving pressure, which was recently identified as a major risk factor for mortality in ARDS patients [6].

VT reduction to < 6 ml/kg to achieve very low P_plat_ induces severe hypercapnia, which raises intracranial pressure, causes pulmonary hypertension, decreases myocardial contractility, reduces renal blood flow, and releases endogenous catecholamines [7, 8]. This strategy is therefore not possible for most ARDS patients on conventional mechanical ventilation (MV) [9]. Extracorporeal carbon-dioxide removal (ECCO_2_R) may be used to achieve VT < 6 ml/kg, thereby lowering P_plat_ and driving pressure in this setting [10–13]. However, the ability to decrease MV intensity with these ECCO_2_R devices, especially those based on a renal replacement therapy (RRT) platform, are limited to animal [14] or single-center [11, 15–17] studies.

The aim of this prospective, multicenter study was to evaluate the safety and feasibility of a low-flow ECCO_2_R device managed by an RRT platform (PrismaLung®; Gambro-Baxter, Meyzieu, France) to enable very low tidal volume ventilation in patients with mild-to-moderate ARDS.

## Methods

### Study design and procedure

This pilot study was conducted during a 14-month period (March 2016–June 2017) in five medical and surgical intensive care units (ICUs) experienced in the care of ARDS patients and use of extracorporeal gas-exchange devices. It was approved by appropriate legal and ethics authorities (Comité de Protection des Personnes Ile-de-France 6, Paris, France; no. 15.1026). The clinical trial protocol was registered with www.clinicaltrials.gov (ClinicalTrials.gov identifier: NCT02606240).

### Patients

As predefined, 20 consecutive patients were included. Inclusion criteria were: mild-to-moderate ARDS according to the Berlin definition [18], 100 mmHg < partial alveolar oxygen pressure/fraction of inspired oxygen (PaO_2_/FiO_2_) < 300 mmHg with positive end-expiratory pressure (PEEP) > 5 cmH_2_O on MV expected to last > 24 h; and bilateral opacities on chest imaging. Exclusion criteria were: age < 18 years, pregnancy, patients with decompensated heart failure or acute coronary syndrome, severe chronic-obstructive pulmonary disease, respiratory acidosis with partial pressure of blood carbon dioxide (PCO_2_)> 80 mmHg, acute brain injury, severe liver insufficiency (Child–Pugh scores > 7) or fulminant hepatic failure, heparin-induced thrombocytopenia, systemic anticoagulation contraindicated, decision to limit therapeutic interventions, catheter access to femoral vein or jugular vein impossible, pneumothorax, and platelet count < 50 G/L.

### ECCO_2_R system

ECCO_2_R was provided by a low-flow, standalone (without concomitant RRT), CO_2_-removal device (Prismalung®; Gambro-Baxter) integrated into the Prismaflex® platform (Gambro-Baxter). The polymethylpentene, hollow fiber, gas-exchanger membrane (surface area 0.32 m^2^) was connected to the extracorporeal circuit, with standard tubes and a Luer-Lock system. A 13-Fr hemodialysis venous catheter (Gamcath™®; Gambro-Baxter) was aseptically and percutaneously inserted under ultrasonography guidance into the right jugular (15 cm) or the femoral (25 cm) vein after an unfractionated heparin bolus (80 IU/kg). Systemic heparinization was started after catheter insertion aiming for an activated partial thromboplastin time ratio (aPTTr) 1.5–2.0× that of the control. Blood was drawn from the superior vena cava and reinjected into the right atrium through the distal lumen. The Prismaflex® device monitored continuous venous, arterial line, and filter pressures.

### Study protocol

Patients were sedated, paralyzed, and ventilated in accordance with the EXPRESS trial protocol [19]: VT at 6 ml/kg of predicted body weight (PBW); PEEP set to achieve P_plat_ of 28–30 cmH_2_O; and respiratory rate (RR) set at 20–35 breaths/min to maintain approximately the same minute ventilation as before study initiation. After priming, the Prismaflex® device was connected to the patient and extracorporeal blood flow was progressively increased to 400–450 ml/min. Sweep-gas flow through the membrane remained at 0 L/min during this phase such that, initially, no CO_2_ was removed.

Following a 2-h run-in time, VT was gradually reduced from 6 to 5, 4.5, and 4 ml/kg PBW every 30 min and PEEP adjusted to reach 23 ≤ P_plat_ ≤ 25 cmH_2_O. At each VT level: if arterial PaCO_2_ rose by > 20% from the baseline PaCO_2_ obtained at 6 ml/kg, the sweep-gas flow through the ECCO_2_R device was switched on with 100% oxygen at 10 L/min; if PaCO_2_ was maintained within ± 20% of baseline PaCO_2_, VT was gradually decreased to a minimum of 4 ml/kg_;_ and if PaCO_2_ remained < 20% at 4 ml/kg PaCO_2_ under the aforementioned ECCO_2_R settings, the RR could be decreased to 15–18 breaths/min. On the other hand, the RR could also be increased up to 35 breaths/min to maintain PaCO_2_ within the targeted range. If undesirable hypercapnia/acidosis persisted (i.e., > 20% 6 ml/kg PaCO_2_), VT was reincreased to the previous step level. Refractory hypoxemia and/or hypercapnia could be managed, at the attending physician’s discretion, with nitric oxide, prone positioning, and/or venovenous extracorporeal membrane oxygenation (ECMO).

The ECCO_2_R-facilitated very low tidal volume ventilation strategy was continued for at least 24 h. The potential for weaning off very low tidal volume ventilation and ECCO_2_R was assessed daily if PaO_2_/FiO_2_ > 200 by setting MV according to conventional ARDSnet settings (VT = 6 ml/kg, PEEP = 5–10 cmH_2_O, RR = 20–30 breaths/min, FiO_2_ = 40%) and switching off the sweep-gas flow through the ECCO_2_R device. If, under these conditions, the patient remained stable for at least 12 h with P_plat_ < 25 cmH_2_O and PaCO_2_ < 50 mmHg (allowing for RR up to 30–35 breaths/min), the ECCO_2_R device and venous catheter were removed. The manufacturer determined the Prismalung® membrane’s maximum duration to be 72 h.

### Data collection

Ventilator settings (VT, PEEP, RR, P_plat_, minute ventilation, FiO_2_), hemodynamic parameters (mean arterial pressure, heart rate, vasopressor dose) and arterial blood-gas values (pH, PaO_2_, PaCO_2,_ HCO_3_^−^, lactate), heparin dose, and aPTTr were collected at baseline, after the run-in-time, 30 min after every VT reduction, and at least twice a day during the subsequent days on ECCO_2_R. Blood-chemistry determinations were obtained daily. Respiratory-system compliance and driving pressure were calculated according to the standard formulas [6, 20]. CO_2_ clearance by ECCO_2_R (ml/min) during the first 24 h was calculated as follows [17]:

(CtCO_2PRE_ – CtCO_2POST_) × 22.4 × ECCO_2_R blood flow / 1000,

where CtCO_2PRE_ and CtCO_2POST_ were the pre and post oxygenator blood CO_2_ content, and CtCO_2_ (mmol/l) = (0.0307 × PCO_2_) + HCO_3_^−^_actual_.

Serious adverse events (SAEs) were prospectively defined as: any event that is fatal or immediately life-threatening, permanently disabling, severely incapacitating, or requires prolonged hospitalization; OR any event that may jeopardize the patient and requires medical or surgical intervention to prevent one of these outcomes; AND any event that the attending physician perceives might be directly related to enrollment in the clinical trial. An AE was defined as: study related when it could be attributed to a study procedure and could readily have been produced by the study procedure; or nonstudy related when it was related primarily to the underlying disease or to ARDS and its sequelae. Other AEs not fulfilling this definition were recorded in the patients’ case-report forms. After ECCO_2_R discontinuation, subjects were monitored for AEs until hospital discharge or day 8 post enrollment, whichever occurred first.

### Statistical analyses

Statistical analysis was performed by one-way analysis of variance (ANOVA) for repeated measures, followed by a Bonferroni post-hoc test for comparison between different times. Results are expressed as mean ± SD and *p* < 0.05 defined statistical significance. Analyses were computed with StatView v5.0 (SAS Institute Inc., Cary, NC, USA) and SPSS v22 (SPSS Inc., Chicago, IL, USA) software.

## Results

**Fig. 1:**
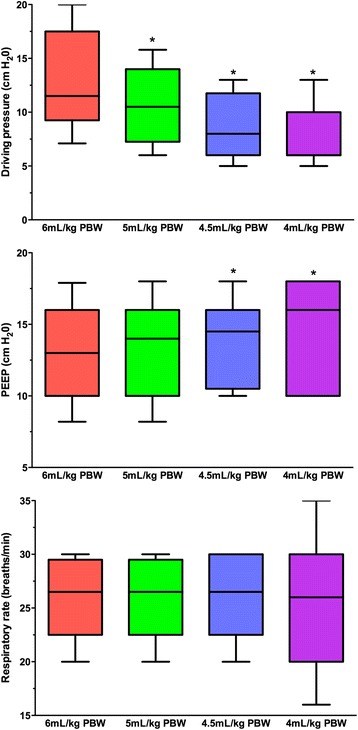
Evolution of driving pressure, PEEP, and respiratory rate when tidal volume reduced on ECCO_2_R. Horizontal lines, median; lower and upper box limits, 25th–75th percentiles; T-bars, 10th–90th percentiles. *p < 0.05 vs 6 ml/kg tidal volume step. PBW predicted body weight, PEEP positive end-expiratory pressure

Twenty patients with mild (*n* = 8) or moderate ARDS (*n* = 12) were included; 18 underwent jugular cannulation. Patients’ baseline characteristics are reported in Table 1. Neuromuscular blockade, nitric oxide, and prone positioning were applied before inclusion to 16, 9, and 8 patients, respectively. Ventilator settings during the VT reduction phase are presented in Table 2 and Fig. 1. At baseline, all patients received protective ventilation with VT set at 6.10 ± 0.30 ml/kg PBW and PEEP at 13.4 ± 3.6 cmH_2_O. VT was gradually lowered to 4 ml/kg for all but one patient (who remained at the 4.5 ml/kg step because PaCO_2_ increased > 20% from baseline at the 4.5 ml/kg step despite ECCO_2_R; see Table 2). While P_plat_ was decreased < 25 cmH_2_O with VT reduction to 4 ml/kg, PEEP was significantly increased from 13.4 ± 3.6 cmH_2_O at baseline to 15.0 ± 3.4 cmH_2_O, according to the very low tidal volume ventilation strategy. As a result, the driving pressure was reduced from 13.0 ± 4.8 to 7.9 ± 3.2 cmH_2_O (*p* < 0.05). Mean PaCO_2_ increased from 43 ± 8 to 53 ± 9 mmHg and mean pH decreased from 7.39 ± 0.1 to 7.32 ± 0.10 from baseline to 4 ml/kg VT, while RR was not modified. The mean CO_2_-removal rate was 51 ± 26 ml/min with 421 ± 40 ml/min blood flow and sweep-gas flow set at 10 ± 0.3 L/min. Importantly, VT and driving pressure reductions with ECCO_2_R were not accompanied by significant changes of PaO_2_/FiO_2_, respiratory-system compliance, and hemodynamic status (Table 2). In the 24 h following ECCO_2_R initiation, nitric oxide was applied to four patients, of whom two also received prone positioning. No patients required ECMO for worsening hypoxemia while receiving very low tidal volume ventilation.

**Table 1:** Baseline characteristics of the 20 patients

Characteristic	Value
Sex (male/female)	11/9
Age (years)	60 ± 12
Body mass index (kg/m^2^)	30 ± 7
Medical/surgical	14/6
SAPS II	56 ± 21
SOFA score at ECCO_2_R insertion	9.3 ± 4.3
Pulmonary ARDS risk factor	
Community-acquired pneumonia	5 (25)
Nosocomial pneumonia	6 (30)
Inhalation pneumonia	5 (25)
Nonpulmonary ARDS risk factor	
Pancreatitis	2 (10)
Other	2 (10)
Pre-ECCO_2_R adjuvant therapy	
Neuromuscular blockade	16 (80)
Prone positioning	8 (40)
Nitric oxide	9 (45)
Recruitment maneuvers	0 (0)
ECMO	0 (0)
Time from intubation to ECCO_2_R initiation (days)	4 (2–7)
Outcome	
Mechanical ventilation duration (days)	13 (9–38)
ICU length of stay (days)	18 (14–41)
Day-28 mortality	3 (15)

Data presented as *n* (%), mean ± standard deviation, or median (25–75% interquartile range)

*ARDS* acute respiratory distress syndrome, *ECCO*_*2*_*R* extracorporeal carbon-dioxide removal, *ECMO* extracorporeal membrane oxygenation, *ICU* intensive care unit, *SAPS* Simplified Acute Physiology Score, *SOFA* Sequential Organ-Failure Assessment

**Table 2:** Time course of ventilation parameters during the run-in phase

Parameter	Baseline (*n* = 20)	VT 5 ml/kg (*n* = 20)	VT 4.5 ml/kg (*n* = 20)	VT 4 ml/kg (*n* = 19)^a^
Ventilation variable				
VT (ml/kg PBW)^b^	6.10 ± 0.30	5.04 ± 0.22^c^	4.49 ± 0.12^c^	3.98 ± 0.18^c^
RR (breaths/min)	26 ± 4	26 ± 4	26 ± 4	25 ± 6
PEEP (cmH_2_O)^b^	13.4 ± 3.6	13.4 ± 3.3	14.4 ± 3.3v	15.0 ± 3.4
P_plat_ (cmH_2_O)^b^	26.3 ± 3.5	24.1 ± 3.0^c^	23.3 ± 2.8^c^	22.8 ± 2.6^c^
Driving pressure (cmH_2_O)^b^	13.0 ± 4.8	10.7 ± 3.8v	8.9 ± 3.3v	7.9 ± 3.2^c^
Compliance (ml/cmH_2_O)	33.8 ± 14.2	33.6 ± 12.7	36.0 ± 13.3	36.9 ± 13.4
PaO_2_/FiO_2_	188 ± 75	192 ± 80	191 ± 71	184 ± 67
Blood gases				
pH^b^	7.39 ± 0.1	7.36 ± 0.10	7.34 ± 0.10^c^	7.32 ± 0.10^c^
PaO_2_ (mmHg)	96 ± 36	93 ± 30	96 ± 24	89 ± 19
PaCO_2_ (mmHg)^b^	43 ± 8	46 ± 7	49 ± 9^c^	53 ± 9^c^
HCO_3_ (mmol/L)	26 ± 4	26 ± 4	27 ± 5	27 ± 4
Lactate (mmol/L)	1.4 ± 0.6	1.2 ± 0.4	1.2 ± 0.5	1.2 ± 0.4
Patients on ECCO_2_R, *n*^d^	–	7	14	19
Patients with PaCO_2_ > 50 mmHg	2	9	9	11
ECCO_2_R				
Blood flow (ml/min)	–	424 ± 39	425 ± 38	421 ± 40
Sweep-gas flow (L/min)	–	10 ± 0.3	10 ± 0.3	10 ± 0.3
CO_2_ removal (ml/min)	–	–	–	51 ± 26
Hemodynamic				
Mean arterial pressure (mmHg)	76 ± 11	79 ± 20	76 ± 12	77 ± 19
Heart rate (beats/min)	86 ± 15	85 ± 13	85 ± 14	83 ± 15
Patients on norepinephrine	9	9	9	10
Norepinephrine dose (μg/kg/min)	0.61 ± 1.10	0.55 ± 1.00	0.55 ± 0.99	0.50 ± 0.97

Values presented as mean ± standard deviation or *n* (%)

*ECCO*_*2*_*R* extracorporeal carbon-dioxide removal, *FiO*_*2*_ fraction of inspired oxygen, *HCO*_*3*_ bicarbonate, *PaCO*_*2*_ partial alveolar carbon dioxide pressure, *PaO*_*2*_ partial alveolar oxygen pressure, *PBW* predicted body weight, *PEEP* end-expiratory positive pressure, *P*_*plat*_ plateau pressure, *RR* respiratory rate, *VT* tidal volume

^a^One patient’s PaCO_2_ increased > 20% at the VT 4.5 ml/kg step and did not undergo further VT reduction

^b^*p* < 0.05, analysis of variance

^c^*p* < 0.05 vs baseline

^d^ECCO2R initiated according to the study protocol when patients had a 20% increase in PaCO_2_ from baseline following VT decrease

Operational characteristics of the ECCO_2_R device recorded in the hour following therapy initiation, including access, return, and filter pressures, are presented in Table 3. Overall mean duration of ECCO_2_R was 31 ± 21 h. It was continued up to 41 ± 24 h until weaning because of improved respiratory condition for 10 patients and was stopped early because of ECCO_2_R-membrane clotting for 10 patients after 20 ± 10 h. The mean daily heparin dose was 19,900 ± 7710 IU/24 h and the mean aPTTr was 1.8 ± 0.6. No cannulation-related complication occurred. One patient suffered a nonfatal cardiac arrest while on ECCO_2_R but this was unrelated to the device. Other AEs included two mild hemoptyses that resolved rapidly without embolization and were not related to heparin overdose. The overall day-28 mortality was 15%.

**Table 3:** Operational characteristics of extracorporeal carbon-dioxide removal for the 20 patients with acute respiratory distress syndrome

Characteristic	Value
Blood flow (ml/min)^a^	421 ± 42
Time of utilization (h)	30.6 ± 21.0
Access pressure (mmHg)^a^	− 145 ± 14
Filter pressure (mmHg)^a^	301 ± 19
Return pressure (mmHg)^a^	154 ± 21
Heparin bolus at insertion (IU)	3100 ± 1330
Heparin (IU/kg/24 h)	230 ± 78
Activated partial thromboplastin time ratio	1.8 ± 0.6
Serious adverse event^b^	
Nonfatal cardiac arrest	1 (5)
Study-related adverse event	
Mild hemoptysis resolved with stopping anticoagulation^c^	2 (10)
Membrane clotting	10 (50)
Time it occurred (h)	20.0 ± 9.7

Values presented as mean ± standard deviation or *n* (%)

^a^Recorded in the hour following initiation of extracorporeal carbon-dioxide removal

^b^Not device related

^c^Resolved without embolization and not related to heparin overdose

## Discussion

The results of this multicenter pilot study showed that a low-flow ECCO_2_R device managed by the RRT platform easily and safely enabled very low tidal volume ventilation with highly significant decreases of P_plat_ and driving pressure in patients with mild-to-moderate ARDS.

Total energy determinants (i.e., mechanical power) are transmitted to the lung by the ventilator-generated volume, pressure, flow, and RR [21]. Decreasing MV intensity and, thereby limiting VILI, requires a diminution of the total mechanical power transferred to the lung [21]. More than 15 years ago, it was demonstrated that volume-limited ventilation with 6 ml/kg PBW significantly lowered ARDS-associated mortality [3]. However, recent data suggested that some ARDS patients are exposed to hyperinflation and overdistension, despite protective ventilation with 6 ml/kg VT and P_plat_ limited to < 30 cmH_2_O. Pertinently, Hager et al. [5] demonstrated that lower P_plat_ was associated with less mortality and that no safe low P_plat_ threshold could be identified in patients with acute lung injury/ARDS. Furthermore, based on a prospective series of 485 consecutive patients with acute lung injury on MV, Needham et al. [22] showed that, compared with a mean VT < 6.5 ml/kg PBW, the adjusted hazard ratios for 2-year mortality for a mean VT of 6.5–8.5 ml/kg PBW was 1.59 (95% CI 1.19–2.14; *p* = 0.001). Amato et al. [6] recently reported that, in addition to VT, P_plat_, and PEEP, normalizing VT to respiratory-system compliance (C_rs_) and using a ratio as an index indicating the “functional” size of the lung might provide a better predictor of ARDS patients’ outcomes than VT alone. That ratio, termed the driving pressure (*ΔP* = VT */* C_rs_), can be routinely calculated as the P_plat_ – PEEP for patients who are not making inspiratory efforts. Their analyses indicated that VT reductions or PEEP increases driven by random treatment-group assignment were beneficial only when associated with *ΔP* decreases and that no other ventilation variable had such a mediating effect on mortality [6]. More recently, lower *ΔP* was also was also associated with lower ARDS-patient mortality in the large LUNG-SAFE cohort [23].

Furthermore, reducing VT to 4 ml/kg PBW in patients already receiving protective ventilation was associated with less inflammatory and morphological signs of VILI in ARDS patients [11]. This particular study used ECCO_2_R to mitigate the respiratory acidosis, and its potent deleterious effects [7, 8, 24], which developed in all patients receiving VT < 6 ml/kg IBW [10, 11]. Results based on previous case series using various ECCO_2_R devices showed the feasibility of this strategy in ARDS patients, although AEs (e.g., cannulation-related accidents, limb ischemia, hemorrhage, hemolysis, infections, pump malfunction, membrane clotting, and catheter displacement) were reported [10, 11, 25–28].

Our results demonstrated that this strategy might be safely, efficiently, and easily applied to ARDS patients in most ICUs worldwide, because it did not require specific or large venous accesses and the RTT platform we used is widely available with minimal modification of existing devices and a simple software update. ECCO_2_R with this RRT platform has indeed obtained promising results in animals [14]. By decreasing VT to 4 ml/kg PBW and adjusting PEEP to a lower P_plat_ target of 23–25 cmH_2_O, we were able to drastically decrease the driving pressure to < 8 cmH_2_O, which might mean less VILI and ultimately fewer deaths [6]. Importantly, we did not observe worsening oxygenation that might have indicated lung derecruitment following the mean airway-pressure decrease [28, 29], although some patients with the most severe forms of ARDS continued to receive nitric oxide or prone positioning following ECCO2R initiation. The PEEP increase resulting from the ventilator strategy used might have counterbalanced that potential hazard [11, 13, 28, 30]. The absence of worsening oxygenation also argues against alveoli nitrogen washout and potential absorption atelectasis, which is less likely to occur in low-flow ECCO2R than during high-flow VV-ECMO.

Several limitations of our work should be addressed. First, because our population was small, this study should only be considered “a proof-of-concept” demonstrating the feasibility and safety of the strategy tested. We cannot rule out that there is still a substantial risk of adverse events that could have been missed in this small study. Second, our population included only patients with mild or moderate ARDS. Because severe ARDS patients might experience greater PaCO_2_ increases and more severe hypoxemia after VT reduction, the Prismalung® performance remains unknown in this context. Third, to achieve VT reduction down to 4 ml/kg in a larger population of patients without the risks of inducing major PaCO_2_ increases not compensated by the low-flow ECCO_2_R device, we also applied the modified EXPRESS strategy to patients with mild ARDS. Because of higher PEEP settings in this population of patients with higher compliance, it should be acknowledged that this approach may induce overdistension despite lowering the driving pressure. In addition, potential benefits of a very low tidal volume ventilation strategy have only been suggested in moderate-to-severe ARDS patients until now [10, 11]. Fourth, we did not evaluate lung morphological and inflammatory markers or the long-term clinical efficacy of the device. Fifth, due to its smaller membrane oxygenator surface, the CO_2_-removal rate of the Prismalung® was lower than those reported in other studies using similar blood flows [17, 28], explaining the gradual increase in PaCO_2_ observed during the VT reduction phase. This mild respiratory acidosis might have been corrected by increasing the RR, at the expense of an increase in mechanical power. Indeed, the physicians treating these patients decided to tolerate this mild acidosis, as recent data also suggest an increased RR might be associated with a poorer ARDS prognosis [31]. Lastly, despite our heparin-infusion protocol that also included a bolus at catheter insertion, 50% of the treated patients experienced membrane clotting before the end of the experimental protocol, as reported previously for other case series given low-flow ECCO_2_R [11, 15]. This technical downside deserves further investigations as it could limit the efficacy and impact the cost–benefit ratio of the device. The development of regional circuit anticoagulation strategies, with blood flows up to 500 ml/min, might enhance ECCO_2_R membrane duration, as was the case for RRT hemofilters [32].

## Conclusions

In summary, our pilot study findings demonstrated that a low-flow ECCO_2_R device managed by an RRT platform enabled very low tidal volume ventilation with moderate increase in PaCO_2_ in patients with mild-to-moderate ARDS. This less-invasive ECCO_2_R technique was easily and safely implemented. However, before this technique can be widely disseminated, more data are needed to demonstrate the clinical benefit of VT, P_plat_, and driving pressure reductions rendered possible by ECCO_2_R [33]. The ongoing international randomized clinical trials SUPERNOVA (ClinicalTrials.gov identifier: NCT02282657) and REST (Clinical-Trials.gov identifier: NCT02654327) focused on moderate ARDS will help clarify this potential.

## Abbreviations

AE: Adverse event
ANOVA: One-way analysis of variance
aPTTr: Activated partial thromboplastin time ratio
ARDS: Acute respiratory distress syndrome
C_rs_: Respiratory-system compliance
ECCO_2_R: Extracorporeal carbon-dioxide removal
ECMO: Extracorporeal membrane oxygenation
FiO_2_: Fraction of inspired oxygen
MV: Mechanical ventilation
PaO_2_: Partial alveolar oxygen pressure
PBW: Predicted body weight
PEEP: Positive end-expiratory pressure
P_plat_: Plateau pressure
RR: Respiratory rate
RRT: Renal replacement therapy
SAE: Severe adverse event
VILI: Ventilator-induced lung injury
VT: Tidal volume
